# Current Trend of Artificial Intelligence Patents in Digital Pathology: A Systematic Evaluation of the Patent Landscape

**DOI:** 10.3390/cancers14102400

**Published:** 2022-05-13

**Authors:** Muhammad Joan Ailia, Nishant Thakur, Jamshid Abdul-Ghafar, Chan Kwon Jung, Kwangil Yim, Yosep Chong

**Affiliations:** Department of Hospital Pathology, College of Medicine, The Catholic University of Korea, Seoul 06591, Korea; vetjoan@gmail.com (M.J.A.); nishantbiotech2014@gmail.com (N.T.); jamshid@catholic.ac.kr (J.A.-G.); ckjung@catholic.ac.kr (C.K.J.); kangse_manse@catholic.ac.kr (K.Y.)

**Keywords:** artificial intelligence, deep learning, digital pathology, intellectual property, patents

## Abstract

**Simple Summary:**

The combination of digital pathology (DP) with artificial intelligence (AI) offers faster, more accurate, and more comprehensive diagnoses, resulting in more precise individualized treatment. As this technology is constantly evolving, it is critical to understand the current state of AI applications in DP. Thus, it is necessary to analyze AI patent applications, assignees, and leaders in the field. In this study, five major patent databases, namely, those of the USPTO, EPO, KIPO, JPO, and CNIPA, were searched using key phrases, such as DP, AI, machine learning, and deep learning, and 523 patents were shortlisted based on the inclusion criteria. Our data demonstrated that the key areas of the patents were whole-slide imaging, segmentation, classification, and detection. In the past five years, an increasing trend in patent filing has been observed, mainly in a few prominent countries, with a focus on the digitization of pathological images and AI technologies that support the critical role of pathologists.

**Abstract:**

The integration of digital pathology (DP) with artificial intelligence (AI) enables faster, more accurate, and thorough diagnoses, leading to more precise personalized treatment. As technology is advancing rapidly, it is critical to understand the current state of AI applications in DP. Therefore, a patent analysis of AI in DP is required to assess the application and publication trends, major assignees, and leaders in the field. We searched five major patent databases, namely, those of the USPTO, EPO, KIPO, JPO, and CNIPA, from 1974 to 2021, using keywords such as DP, AI, machine learning, and deep learning. We discovered 6284 patents, 523 of which were used for trend analyses on time series, international distribution, top assignees; word cloud analysis; and subject category analyses. Patent filing and publication have increased exponentially over the past five years. The United States has published the most patents, followed by China and South Korea (248, 117, and 48, respectively). The top assignees were Paige.AI, Inc. (New York City, NY, USA) and Siemens, Inc. (Munich, Germany) The primary areas were whole-slide imaging, segmentation, classification, and detection. Based on these findings, we expect a surge in DP and AI patent applications focusing on the digitalization of pathological images and AI technologies that support the vital role of pathologists.

## 1. Introduction

Digital pathology (DP) is an emerging medical technology that can be used as a platform for artificial intelligence (AI) to provide faster, more accurate, and more thorough diagnoses, leading to more precisely tailored therapy [1,2]. Traditionally, a pathological diagnosis based on microscopic examination using glass slide samples has been considered the gold standard for cancer diagnosis. However, in recent years, enormous changes have occurred following the introduction of the digitalization technique using a whole-slide scanner, in conjunction with the recent advances in AI-based image analysis [2,3]. During the past several years, many AI applications have exhibited promising results in cancer studies using DP, including the segmentation of carcinoma foci, detection of lymph node metastasis, counting of tumor cells, and prediction of genetic mutations [4,5,6,7]. Most studies have demonstrated comparable or even slightly superior results to those of conventional microscopic diagnosis in terms of diagnostic accuracy. As these AI models are being developed to reduce the time and labor of pathologists, they can reduce the turnaround time of pathological examination. Such AI models can provide second opinions to pathologists in a supportive manner, which is helpful for a more comprehensive diagnosis [8,9].

Owing to the rapid growth of AI in DP, patent analysis is useful for comprehending the current state of technology in terms of appraisal and future economic value [10,11]. The global market for DP systems is expected to reach USD 1.4 billion by 2027, with a compound annual growth rate of 13.9% [8]. The State Council of China issued a growth strategy in 2017, with the goal of making China the global leader in AI by 2030 [9]. Therefore, substantial focus has been placed on AI development in DP. In 2017, Jiang et al. reviewed AI in the healthcare sector and concluded that AI-powered technology would be more widely available and applied in future healthcare setups [12]. In a review paper on the patent landscape of DP published in 2014, a research group from the University of Pittsburgh concluded that the number of such patents had quadrupled over the past decade and that this would continue in the following years [13]. Another study by Abadi et al. demonstrated that AI patent registrations with the United States Patent and Trademark Office (USPTO) have increased steadily over time [10]. However, there remains a lack of data on the patent landscape of AI applications in DP that considers the global trends, primary databases, and progress achieved within the previous decade, particularly the past five years.

Almost every region has a patent office that is responsible for registering and protecting local innovations under patent law. Major economies and innovator countries have a broader range of inventions and scope for patent protection. As a result, they frequently collaborate, such as in the IP5 collaboration, which is comprised of the five major patent offices in the world: (i) the USPTO; (ii) the European Patent Office (EPO); (iii) the Korean Intellectual Property Office (KIPO); (iv) the Japan Patent Office (JPO); and (v) the People’s Republic of China’s National Intellectual Property Administration (CNIPA). Together, the IP5 Offices process about 80% of all patent applications worldwide and 95% of all work conducted under the Patent Cooperation Treaty (PCT) with a vision of “Patent harmonization of practices and procedures, enhanced work-sharing, high-quality and timely search and examination results, and seamless access to patent information to promote an efficient, cost-effective and user-friendly inter-national patent landscape”. Thus, we conducted a systematic review of the worldwide landscape of AI-related DP patents utilizing five major databases, namely, those of the USPTO, KIPO, EPO, JPO, and CNIPA, in order to acquire a better understanding of AI’s current position in DP and to gain insight into its future trajectory.

## 2. Materials and Methods

### 2.1. Database Search

This study was performed in accordance with the Preferred Reporting Items for Systematic Reviews and Meta-Analyses (PRISMA) and was approved by the Institutional Review Board of the Catholic University of Korea, College of Medicine (UC21ZISI0053). Five databases (the USPTO, KIPO, EPO, CNIPA, and JPO databases) were searched using keywords relating to DP and AI up until May 2021. The searched queries included the terms: “Digital pathology”, “Deep learning”, “Artificial intelligence”, “Telepathology”, “Computer assisted diagnosis”, “Machine learning”, “AI diagnosis”, “Deep learning diagnosis”, “Digital imaging”, “Pathology”, “Whole slide scanner”, “Whole slide imaging”, “Digital microscopy”, “Digital image analysis”, “Image analysis”, and possible combinations thereof.

### 2.2. Data Search and Retrieval

**Figure 1:**
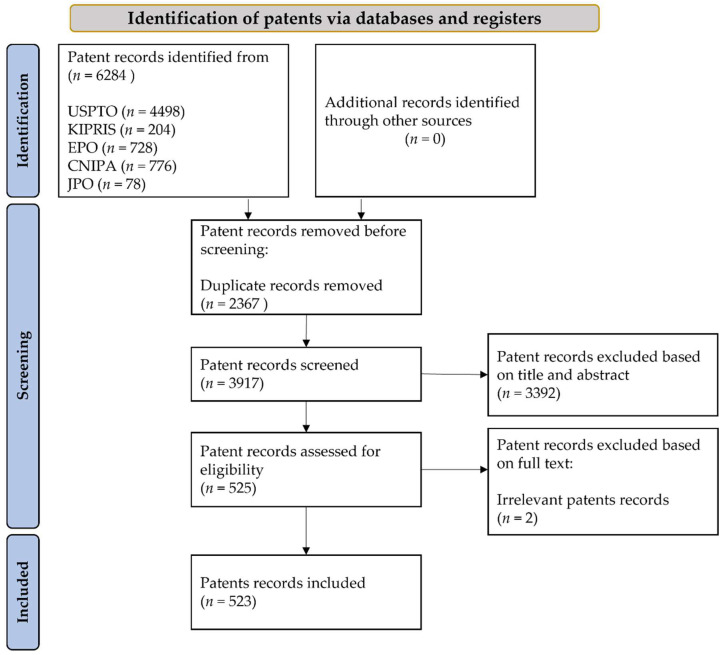
PRISMA diagram for patent search and retrieval.

The search and review processes are summarized in Figure 1. After the initial search, duplicate patents were excluded from the results. The titles and abstracts of the patents were screened by two independent reviewers (M.J.A. and Y.C.). Patents and applications covering technologies relevant to AI involvement in DP as well as those for technologies produced for other disciplines but nevertheless used (“usable”) in DP were included. Thus, the progress and role of AI in the past two decades were examined and classified according to the year, country, and direct or indirect uses of DP.

### 2.3. Data Analysis

The data were analyzed using Microsoft Excel version 2201 (Build 14827.20192) and Python version 3.9.6. The patents were screened and examined based on the title and description of the invention. The dataset was examined using the yearly trend of patent publications, global AI patent applications, top 10 assignees, and inventors to determine the trends of patent registration, nations participating in innovations, key assignees, and inventors. Furthermore, the patents were examined according to topic categories to establish innovation trends and which areas of DP are most significant for AI applications.

## 3. Results

### 3.1. Database Search

A flow diagram of the patent selection process is presented in Figure 1. A total of 6284 patent records were found in the different patent IPO databases, among which 523 patents were selected based on the title and description of the invention (Supplementary s1data.xls).

### 3.2. Global Trend of AI and DP Filing over the Years

**Figure 2:**
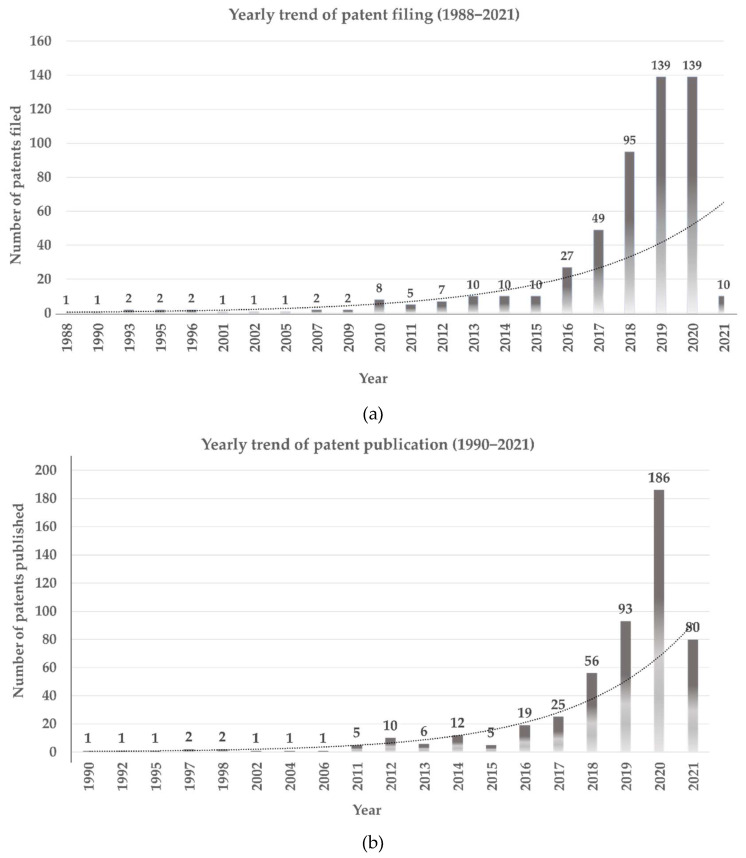
Yearly trends of publication and filing of patents according to AI and DP. (**a**) Patent filing. (**b**) Patent publication.

Our statistics demonstrated an increasing trend in patent applications and publications over the past decade (Figure 2a,b). During the past five years, significant growth in patent filing was observed, with 10 patents filed in 2015 and 139 patents in 2020 (Figure 2a). The number of publications also increased significantly from five to 186 between 2015 and 2020 (Figure 2b).

### 3.3. Top Assignee Countries

**Figure 3:**
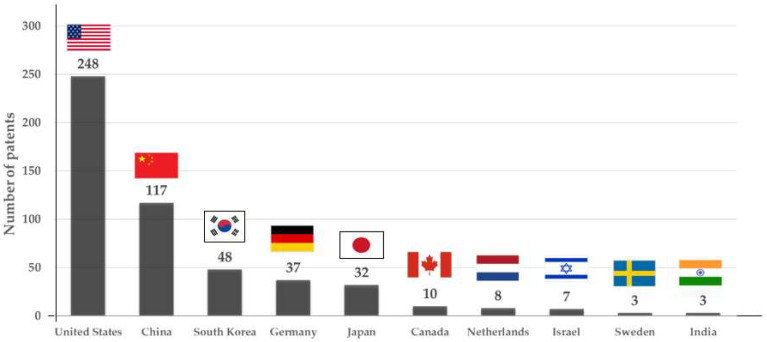
Number of AI patents in DP by country.

Based on our systematic review analysis, we identified only 523 AI-based DP patents that were granted by different IPOs globally from 1990 to 2020. The United States (248 patents) was the top assignee, followed by China (117), South Korea (48), Germany (37), Japan (32), and many other countries (Figure 3). Our data indicate that AI has been a primary focus in most developed countries over the past decade, and the protection of inventions by filing patents has increased over the years. In recent years, more contributions have been made to AI development by other countries, such as India and Sweden, which have only recently been introduced to the market (Figure 3).

### 3.4. Top Assignees

**Figure 4:**
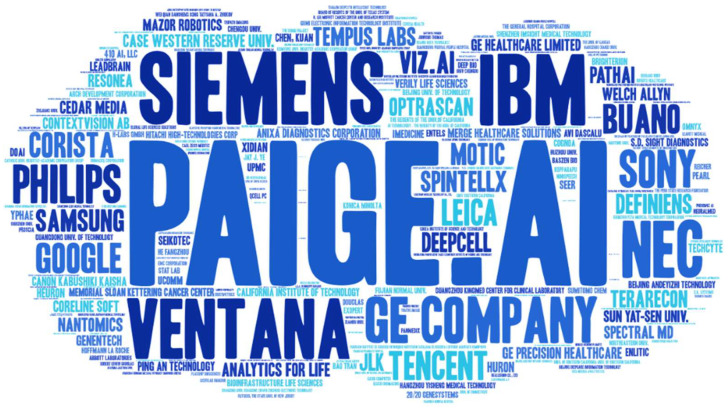
Word cloud analysis for patent assignees.

According to the top 10 assignees based on AI DP patents, most patents were filed by companies, with the exception of Case Western University, from January 1990 to December 2020. Among these, Paige.AI, Inc. (New York City, NY, USA) and Siemens, Inc. (Munich, Germany), which were established by Thomas Fuchs and El-Zehiry Noha, respectively, were the leading companies, with 25 patents each between 2014 and 2020 (Table 1). These companies were not listed before 2009, indicating that the recent development of the field and new emerging companies played a leading role in AI development. Meanwhile word cloud analysis was also performed to graphically represent the contribution of major companies and role players of AI in DP (Figure 4).

### 3.5. Subject Categorization of Patents

**Figure 5:**
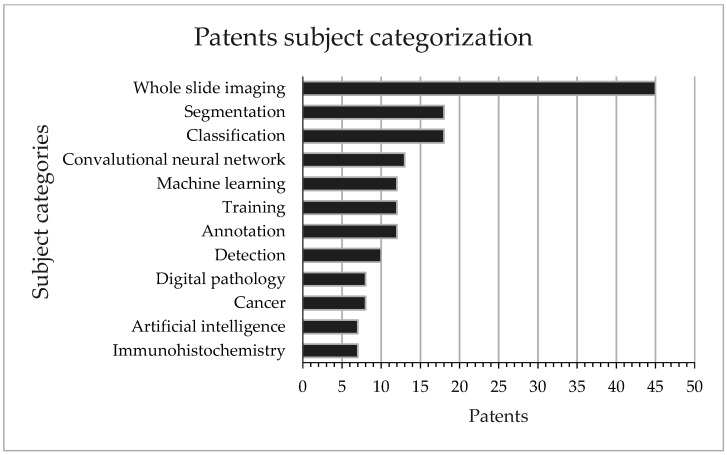
Subject categorization of the patents in AI and DP.

The patents were analyzed based on the titles, abstracts, and full texts and classified based on the keywords used. Considering that artificial intelligence is a broad term with numerous subcategories that intersect and overlap, we categorized the patents according to the principal area of interest of the assignee and devised a system of categories. The primary analysis revealed that most assignees focused on innovation related to whole-slide imaging (WSI), segmentation, classification, convolutional neural networks (CNNs), training, detection, and annotation (Figure 5). Most of these patents covered cases that were directly or indirectly related to tumor detection.

#### 3.5.1. WSI

WSI was one of the top categories of AI-related patents in DP. The Bacus Research Laboratory, Inc. (Elmhurst, IL, USA) was the first to patent WSI technology; they submitted WSI patents in 1997 and 1998 that were awarded in 2000 (patent 6101265) and 2001 (patent 6272235), respectively [14,15]. They described a WSI system for acquiring an image of an entire glass slide and presenting it to a pathologist on a computer monitor. The succeeding patents disclosed several methods for digitally capturing entire glass slides, including linear arrays. The vast majority of these were assigned to Aperio Leica (patents 6711283, 6917696, 7457446, 8055042, 7978894, 8385619, 8755579, 9386211, and 9851550) [16,17,18,19,20,21,22,23,24].

Ventana Medical Systems, Inc. (Tucson, AZ, USA) devised a computer system that can identify the most suitable z-layer in a z-stack or image tiles that are more suitable for cellular-based scoring by a medical professional (10181180) [25]. Another patent from Ventana described a methodology for segmenting images of biological specimens using adaptive classification to divide the material into several categories of tissue regions. Thus, various grids of points (GPs) are marked on the WSI and subsequently classified on a pre-built training database to generate a classification confidence score. GPs with high confidence scores are used to generate an adaptive training database, which is used to reclassify low-confidence GPs (10898222, 10102418) [26,27]. Another patent by Ventana proposed an image analysis technique based on machine learning (ML) for the automatic identification, classification, and counting of objects (e.g., cell nuclei) inside DP tissue slides. The object classifier is trained using the reference sample slides. Thereafter, by means of image segmentation algorithms, the entire slide is divided into a background zone and tissue region. Radial symmetry is used to identify seed locations within prominent colored areas within the tissue data. Based on these seed locations, the facility generates a tessellation, with each region representing a recognized item. Subsequently, the previously trained classifier classifies these items (10176579) [28].

NEC Laboratories America, Inc. (Princeton, NJ) filed a patent for cloud-based DP, in which pathological slides are uploaded and analyzed using one or more nodes each, including one or more processors. Based on the analysis type, the intermediate results are produced and sent to the client device, and the final analysis is issued upon confirmation from the client device (8897537) [29]. The University of South Florida (Tampa, FL, USA) devised automated stereology for determining tissue characteristics, which can capture a z-stack of images of a three-dimensional (3D) object; construct extended depth of field (EDF) images from the z-stacks of images; perform segmentation operations on the EDF images, such as estimating a Gaussian mixture model and performing morphological operations, watershed segmentation, Voronoi diagram construction, and boundary smoothing; and determine one or more stereology parameters (11004199) [30].

#### 3.5.2. Segmentation

Segmentation was the second most frequently used category of DP patents by inventors. The first patent on pathological segmentation algorithms was submitted in 1995 by inventors at the Cedars-Sinai Medical Center (patent 5687251) [31]. Since then, several innovators have published numerous patents on pathological segmentation algorithms. TEMPUS Labs, Inc. (Chicago, IL, USA) patented a technique for the AI segmentation of tissue images in which an overlay map is generated using cell detection and tissue classification methods on a digital medical image of a slide. A medical image is received and divided into tiles, following which tile and tissue classifications are performed using multitile analysis by recognizing cell objects in images, splitting images into polygons identifying cell objects, and showing cell classifications (10991097) [32].

Sony Corporation (Tokyo, Japan) suggested that a DP image segmentation task could be separated into at least two subtasks in certain embodiments. The first subtask can be carried out using both bottom-up and top-down analyses to capture local object boundaries and to minimize false positives. In certain embodiments, the improved segmentation findings are used as inputs to a second sub-task that employs a different algorithm to accomplish the ultimate task by combining bottom-up and top-down image processing (8345976) [33]. Ventana Medical Systems, Inc. (Tucson, AZ, USA) proposed a technique for scoring dual ISH images that include foreground segmentation and nucleus ranking. This approach was created to aid in the identification of nuclei that match the requirements for dual ISH scoring within a particular field of view (10475190, 10909687) [34,35].

Optrascan, Inc. (Cupertino, CA, USA) has worked on determining the potential efficacy of immunotherapy approaches, the segmentation of single cells, and an automated slide scanning system with an image acquisition unit. With a facility for 3D image acquisition (10586376) [36], PROSCIA, Inc. (Philadelphia, PA, USA) has provided processors that receive images displaying tissues and quantify them based on segmenting the image into various segments through the processor and then, in each segment, detect various histological components. A network graph is constructed through the processor that includes a number of nodes, each of which corresponds to a histological element. Using the processor, network graph feature measurement is performed to transform the image, an image non-parametric feature is determined, and the nonparametric feature is sorted in a database (10614285) [37]. The current approach is directed at a computer-implemented system and technique for single-channel whole-cell segmentation of a biological sample image. Biological samples may be stained with one or more nonnuclear cell marker stains, and the system and method are designed to segment the image of a biological sample that is labeled with one or more non-nuclear cell marker stains into one or more cells with demarcated nuclei and cytoplasmic regions (10789451) [38].

#### 3.5.3. Classification

The EMC Corporation (Hopkinton, MA, USA) proposed a cluster-based classification system in 2012. The classification is performed in a parallel manner across multiple processing devices using MapReduce processing, so the classification is performed on multiple machines simultaneously using different hardware and software systems (8873836) [39]. NEC Laboratories America, Inc. (Princeton, NJ, USA) devised systems and methods for classifying histological tissues or specimens in two phases. The first phase involves determining the division of features into sets of increasing computational costs and assigning a computational cost to each set. In the second phase, the method applies classifiers to an unknown tissue sample (9224106) [40]. In 2017, the Case Western Reserve University (CWRU) (Cleveland, OH, USA) registered a patent titled “Histomorphometric classifier to predict cardiac failure from whole-slide hematoxylin and eosin-stained images” (10528848). The Memorial Sloan Kettering Cancer Center (New York, NY, USA) directed their efforts at classification systems and techniques for biomedical imaging [41]. A feature classifier can create a plurality of tiles from a biological image. Each tile may represent a segment of the biological image and each score may represent the probability that the associated tile has a characteristic that is indicative of the existence of the disease in question (10810736) [42]. The CWRU suggested quality control for DP slides, whereby embodiments include accessing a set of DP images with an imaging parameter, applying a low-computational cost histology quality control (HistoQC) pipeline to the DP images, and applying a second, different high-computing cost pipeline. Each step determines an artifact-free region of the member of the first or second cohort and classifies it as suitable or unsuitable for downstream computation or diagnostic analysis (10861156) [43]. A registered patent describes embodiments to predict early-stage non-small cell lung cancer (NSCLC) recurrence and includes processors that are configured to access a pathological image of a region of tissue and a radiological image of the region. The embodiments may display a classification or generate a personalized treatment plan based on this classification (10846367) [44]. Similarly, in 2021, an embodiment predictor of early-stage NSCLC recurrence was proposed, in which a classification of the region is determined as likely or unlikely to experience recurrence based on probability. The embodiments include an image acquisition circuit that is configured to access an image of a region of tissue, including a plurality of cellular nuclei, a nuclei detection and segmentation circuit that can detect a member of the plurality, and classification of the member as a tumor-infiltrating lymphocyte (TIL) nucleus or non-TIL nucleus (10956795) [45].

In 2016, Ventana Medical Systems, Inc. (Tucson, AZ, USA) devised systems and methods for automatic field of view selection in immune computation that involve reading images for individual markers from an unmixed multiplex slide or single-stain slides. The heat map of each marker is determined by applying a low-pass filter to the individual marker image channel (10275880) [46]. The CWRU described the methods and apparatus associated with classifying a region of tissue represented in a digitized WSI using iterative gradient-based quasi-Monte Carlo sampling. A prognosis or treatment plan can be provided based on the WSI classification. One example apparatus includes an image acquisition circuit that acquires the WSI of a section of tissue exhibiting cancerous pathology (10049450) [47]. Furthermore, in 2018, they proposed embodiments including an image acquisition circuit that is configured to access an image of a region of tissue exhibiting cancerous pathology. They constructed a nuclear subgraph based on the detected cellular nuclei, in which a node is the nuclear centroid of a cellular nucleus. They generated a conditional random field (CRF) signature that is based, at least in part, on the set of CRF features and the probability that the region will experience cancer progression (10503959) [48]. DR Systems, Inc. (San Diego, CA, USA) provided systems and methods that allow transfer and display rules to be defined based on one or more of several attributes, such as a particular user, site, device, and/or image/series characteristic. The system and methods may include image analysis, image rendering, image transformation, image enhancement, and other aspects to enable efficient and customized reviews of medical images (9934568) [49]. NantOmics, LLC (Culver City, CA, USA) proposed a computer-implemented method for generating at least one shape of a region of interest (ROI) in a digital image. This method includes access to a digital tissue image of a biological sample and the tiling thereof into a collection of image patches. It also involves classification by applying a trained classifier to patch vectors of other patches in the collection of patches, and the other patches are classified as belonging or not belonging to the same class of interest (10769788) [50]. The Memorial Sloan Kettering Cancer Center (New York, NY, USA) devised systems and methods for classifying biomedical images. A feature classifier can generate multiple tiles from a biomedical image. Each tile may correspond to a portion of the biomedical image and each score may indicate the likelihood that the corresponding tile includes a feature that is indicative of the presence of the condition (10445879) [51].

**Figure 6:**
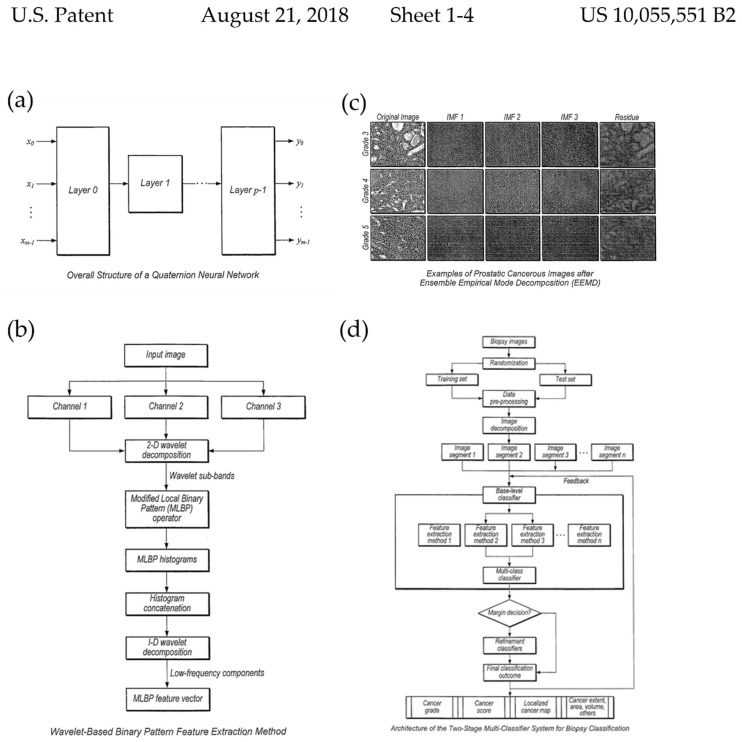
Selected drawings from the patent “Systems and methods for quantitative analysis of histopathology images using multiclassifier ensemble schemes” (patent 10055551) awarded to the Board of Regents of the University of Texas System (Austin, TX, USA) in 2018. (**a**) flow chart diagram depicting an example of a structure of a quaternion neural network (**b**) functional block diagram of an example of wavelet-based binary pattern feature extraction method of the invention (**c**) examples illustrating of prostatic cancerous images after Ensemble Empirical Mode Decomposition (EEMD) (**d**) flow chart diagram depicting an example of an architecture of the two-stage multi-classifier system for biopsy classification. (reproduced from the public database at www.uspto.gov, accessed on 21 October 2021).

The Board of Regents of the University of Texas System (Austin, TX, USA) presented digital images of biopsy slides that can be used to identify cancerous areas in a multistage classification process. Color normalization and segmentation procedures are used to prepare biopsy areas of interest or entire biopsy slides from the digital images. Refinement classifiers are employed in the second stage, which are subsequently trained on a reduced number of classes compared to the first-stage classifiers. A cross-validation approach based on the performance metrics that are obtained through multiple validation rounds is used to alter at least one classifier parameter. One or more digital images of the biopsy slides can be used to conduct multistage classification to identify cancerous regions. Images of diverse bodily tissues that have been scanned are included in the collection. Fuzzy local color transference, deconvolution, the Reinhard technique, histogram matching, and nonlinear color mapping are all methods that are used to normalize image color. Cross-validation is used to segregate the data that are used for training or validation using classification algorithms into two independent groups (Figure 6) (10055551) [52].

#### 3.5.4. CNNs

Many inventors have used a CNN as a base algorithm to fit their deep learning models. Deep learning-based models, particularly CNNs, have recently received considerable attention [53,54]. A CNN can learn multilevel feature hierarchies that are invariant to irrelevant sample perturbations while retaining relevant information [55]. A CNN is composed of numerous convolutional and pooling layers, followed by several fully connected layers. Each convolutional filter corresponds to an output feature map, and a convolutional layer learns a set of convolutional filters that are used to construct the output feature maps. In each feature map, the pooling layer (commonly referred to as max-pooling) summarizes the activities and selects the maximum values over a neighborhood region. The fully connected layer learns higher-level feature representations. The final layer is frequently a softmax layer (fully connected) that outputs the probability that the input patch belongs to a specific category [56].

In 2014, the CWRU (Cleveland, OH, USA) filed a patent for an apparatus for identifying mitosis in breast cancer pathology images containing a logic for acquiring an image of a tissue area and partitioning the image into candidate patches. This method calculates the likelihood of mitosis patches using a handcrafted feature set and a CNN-learned feature set. It trains a classifier based on the weighted averages of the first, second, and third probabilities. Similarly, Leica Biosystems Imaging, Inc. (Vista, CA, USA) devised a patent to recognize cancers in a histological image using a CNN. The CNN is configured using one channel for each of the several tissue classes to be recognized, with at least one class for each nontumorous and tumorous tissue type. Subsequently, the resulting image patches are combined into a probability map that can be displayed alongside or over the histological image (10740896). Sethi et al. devised a system and method for computational pathology using points of interest, in which instructions that are stored in memory and executed by the processor are sent to an imaging device for acquiring images of patient tissue. These instructions force the processor to calculate the disease class scores for patient tissues by equipping the first classifier with a nucleus detector that includes at least one pretrained neural network and a CNN (10573003). In 2019, PHENOMIC AI, Inc. (Toronto, CA, USA) developed a neural network design that includes a CNN and a multiple-instance learning pooling layer. One or more reference cellular phenotypic variables can be predicted from microscopic images of cell populations. Training and testing can be performed directly on raw microscope images in real time without the need for segmentation or cell labeling (10303979). Shenzhen Keya Medical Technology Corporation (Shenzhen, CN) proposed a system for detecting cancer metastasis in a WSI. A fully CNN model and several partially overlapping tiles are used to identify cancer metastasis. Subsequently, a probability detection map is generated that indicates the likelihood that each pixel in the WSI has cancer (10846854). The Regents of the University of California (Oakland, CA, USA) invented a method for determining the optimal image from a sequence of image frames by loading the images into a computer processor and applying a sliding window to the image sequence. The first neural network is trained to categorize the image frames according to their spatial properties and the second neural network is trained to classify each window into two groups. An output is created that indicates the image frame windows that have an ideal image (10909681).

#### 3.5.5. Machine Learning

**Figure 7:**
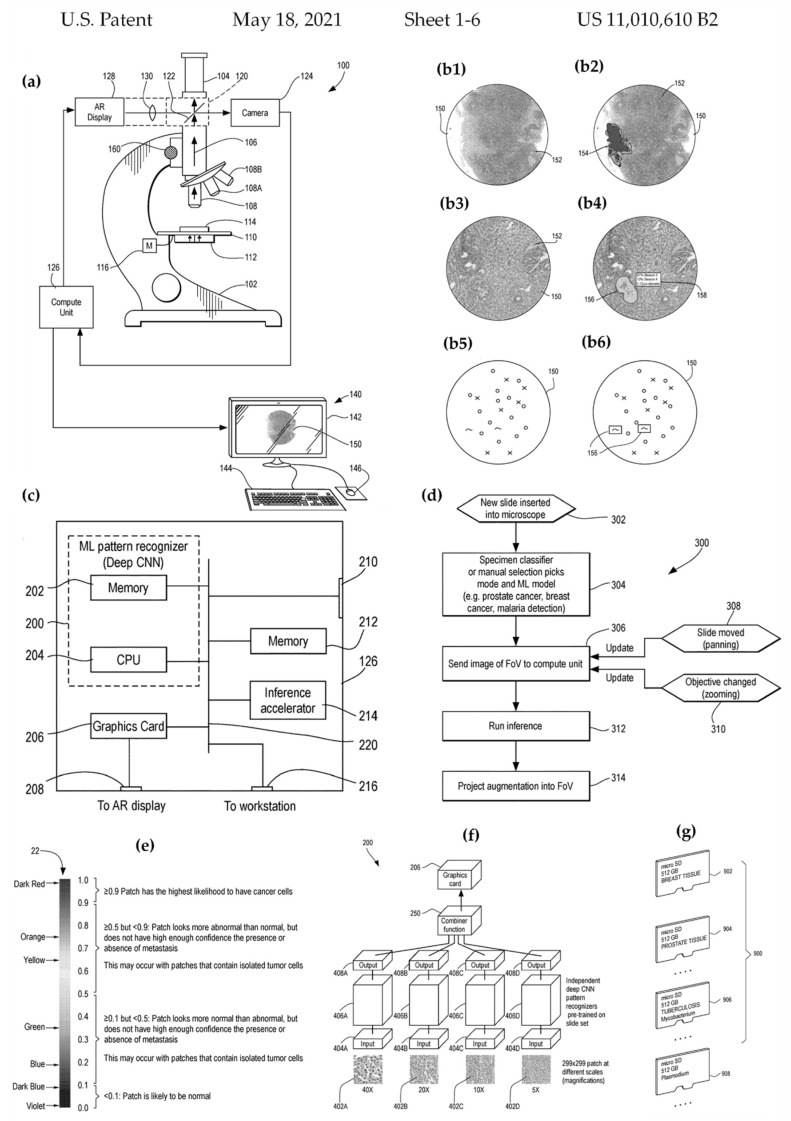
Selected drawings from the patent “Augmented reality microscope for pathology” (patent 11010610) awarded to Google LLC, Mountain View, CA, USA in 2021. (**a**) schematic diagram of an augmented reality microscope system for pathology, (**b1**) illustration of the field of view showing a breast cancer at a given magnification, (**b2**) augmented view seen by the pathologist with an enhancement in the form of a “heat map” superimposed on it (**b3**) Illustration of the field of view of a microscope displaying a prostate cancer specimen at a specific magnification (**b4**) depicts an enhanced view observed by a pathologist using the microscope depicted in figure a, with an outline superimposed on the field of view. The addition also provides a text box including annotations, such as Gleason score grading and tumor size statistics in this case (**b5**) a representation of the field of view of a blood sample under a microscope at low magnification (**b6**) illustrates the field of view of figure f, with the addition of rectangles designating malaria parasites (plasmodium) present in the sample to aid the pathologist in defining the sample (**c**) illustrates an expanded block diagram of figure a computing unit (**d**) a flowchart depicting the work flow of the system depicted in Figure a (**e**) diagram displaying a color coding or scale for reading a heat map (**f**) a representation of a machine learning pattern recognizer as an ensemble of independent deep convolutional neural networks that have been pre-trained on a set of microscope slide images. Each member of the ensemble has been trained at a specific level of magnification (**g**) depicts a set of portable computer storage media, each of which is loaded with code, parameters, and associated data representing an ensemble of independent deep convolutional neural networks trained on a set of microscope slide images for a specific application, such as detection of breast cancer in breast tissue, detection, and characterization of cancer cells in prostate tissue. (Reproduced from the public database at www.uspto.gov, accessed on 21 October 2021).

ML creates predictive models from data to identify patterns or perform tasks, such as regression or classification. ML methods can be classified as two types: supervised and unsupervised learning [57]. In 2010, Sony Corporation (Tokyo, Japan) provided systems and techniques for constructing a multistep image-recognition framework for the classification of digital images. This framework enables a step-by-step approach to model training and image classification problems that require multidimensional ground truths. The first step distinguishes the first image region from the remaining image region, and each subsequent step acts on a portion of the remaining image region of the preceding step (8351676) [58]. In 2012, NEC Laboratories America, Inc. (Princeton, NJ, USA) proposed a method in which an interface layer receives a selection of an image region and a request for analysis, and the selection and request are sent to an interpretation layer for analysis, whereby the image is divided into subsections that are designed for parallel computing to deliver an analytical result with minimal latency. The interpretation layer selects an image and a request for analysis from the interface layer. An execution controller further divides the image into subsections that are designed for parallel computing (8934718) [59]. In 2013, they proposed a computer-implemented technique for completely automated tissue diagnostics that supervises the training of an area of the ROI classifier using only tissue-level labels. This approach identifies the ROIs in a given tissue, extracts feature vectors from each ROI, applies the ROI classifier to each feature vector to obtain a set of probabilities, feeds the probabilities to the tissue classifier, and generates a final diagnosis for the entire tissue (9060685) [60]. In 2021, Robert Edwin Douglas presented a system that generates 3D volumetric datasets using an AI algorithm. The data are subsequently rendered using the mechanical properties that are initially assigned. This process is repeated numerous times as the setup changes. The goal of this cycle is to ensure that each change in the configuration conforms to the nature of the input and the mechanical type of the 3D dataset (10950338) [61]. CORISTA, LLC (Concord, MA, USA) proposed the creation of multiple slides from thin, successive slices of tissue while analyzing DP tissue specimens. The review of numerous WSIs is a challenge because of the lack of uniformity in the images. WSIs are aligned using a multiresolution registration technique, normalized for faster processing, annotated by an expert user, and separated into image patches in several implementations. The image patches may be used to train an ML model to identify characteristics that are helpful for detecting and classifying ROIs in images (10943346) [62]. In 2021, Koninklijke Philips N.V. (Eindhoven, The Netherlands) provided a system and method for learning to annotate objects on one or more scales of a multiscale image using an ML algorithm. A viewing window can be configured using a magnification factor, which determines the scale to be observed, and a spatial-offset parameter. A user can annotate an item in the viewing window manually, which is subsequently used as training data for the ML algorithm (10885392) [63]. In 2018, Techcyte, Inc. (Orem, UT, USA) proposed a disclosure pertaining to the categorization of cells/particles in microscope images using ML. One technique involves entering an image with hidden characteristics into the initial neural network classifier (INNC) of the CNN. The INNC is trained on images that contain the ground truth from out-of-channel mechanisms. The final classification is generated and outputted based on the hidden characteristics that are included in the original image (10552663) [64]. Google LLC (Mountain View, CA, USA) devised a method for facilitating a user to evaluate a slide containing a biological sample using a microscope equipped with an eyepiece, comprising the steps of collecting a digital image of the sample with a camera and identifying the ROI using an ML pattern recognizer. The camera captures a fresh digital image and sends it to the ML pattern recognizer when the magnification or focus of the microscope changes or when the sample is moved relative to the microscope optics. The user can then categorize or describe the sample by superimposing a new enhancement onto that view in near real time. Moreover, the microscope has a motorized stage to support and move the slide relative to the eyepiece, as well as an ML pattern recognizer for performing preliminary identification of the probable ROI in the biological material Figure 7 (11010610) [65].

#### 3.5.6. Training

CWRU (Cleveland, OH, USA) devised a prediction model for cancer recurrence using local co-occurrence of cell morphology (LoCoM), in which the embodiments comprise image acquisition circuitry for identifying and segmenting at least one cellular nucleus in an image of an area of tissue with malignant pathology. Furthermore, they feature circuitry that calculates the chance of cancer progression in a location based on the LoCoM signature, and the region is classified as a progressor or no-progressor based on the probability (10783627) [66]. In 2018, PathAI, Inc. (Boston, MA, USA) developed tools and techniques for training a model to predict the survival time of a patient. This strategy entails gaining access to pathological images that are connected to a cohort of patients who are enrolled in a clinical trial. Each annotated pathological image is correlated with patient survival data. A model is trained based on the survival data and extracted values for the attributes (10650929) [67]. Moreover, they presented a method for predicting tissue features from pathological images. A statistical model that has been trained on various annotated pathological images is employed. Each image of the training pathology contains an annotation outlining the tissue features that are stored on a storage device (11080855) [68]. Enlitic, Inc. (San Francisco, CA, USA) devised the construction of a computer vision model, whereby scans and global labels are used to train the model. To produce the probability matrix data, an inference function that uses the computer vision model on a fresh medical scan is used to determine an image patch probability value for each abnormality class in a series of image patches. These data comprise a collection of global probability values, each representing a specific abnormality class in the new medical scan. These values are subsequently transmitted to a client device as a set of global probabilities (10943681) [69].

Definiens GmbH (Munich, Germany) proposed a method for detecting blurred areas in digital images of stained tissue, which involves the artificial blurring of a learning tile and the training of a pixel classifier to classify each pixel as belonging to either the learning tile or the blurred learning tile. The digital image of a biomarker-stained tissue slice from a cancer patient is separated into tiles. The pixel descriptor of the pixel classifier is constructed by studying and comparing the pixel values of each pixel in the learning area with nearby pixels at predefined offsets from each studied pixel. The descriptor indicates whether a pixel is the most likely part of an unblurred class, such as those in the first subregion, or a blurred class, such as those in the second subregion. A score is calculated using the image objects to represent the degree of cancer malignancy in the tissue slice of the patient (10565479, 10438096) [70,71]. Siemens Aktiengesellschaft (Munich, Germany) developed systems and techniques for image classification that incorporate the acquisition of imaging data from in vivo or excised tissues of a patient during a surgical operation. The imaging data are used to extract the local image characteristics. A vocabulary histogram is produced based on the retrieved local image features. A classifier is trained using a set of validated tissue types in a batch of sample images (10635924) [72].

#### 3.5.7. Detection

In 1998, the University of Pittsburgh (Pittsburgh, PA, USA) optimized and adapted a CAD methodology to determine ROIs in digital images. The optimization is based on the global characteristics of the image. Global image characteristics are assessed for each image in a database of images with an identified ROI and a characteristic image index is constructed using these global image features. The images in the database are classified into several image groups based on their image characteristic index and the CAD scheme is optimized for each image group. After optimizing the CAD scheme for processing a digital image, classification criteria based on the image characteristics are constructed for that image, following which the global image features of the digitized image are determined. After assigning an image rating to the digitized image based on the identified global image characteristics, the image is allocated to an image group based on the image rating. The ROI that is depicted in the image is determined using a detection technique that is tailored to the allocated image group (6278793) [73]. Flagship Biosciences, Inc. (Westminster, CO, USA) devised a novel nanomethod for analyzing tissue specimens based on histology slides that extend far beyond human evaluation and interpretation using an optical microscope. Cells are detected and identified on partial or complete tissue sections, distinct cell populations are defined and characterized within a tissue specimen, and tissues are assessed based on cell population features (9488639) [74]. S.D. Sight Diagnostics Ltd. (Tel Aviv, Israel) developed technology in which a digital camera is programmed to capture images of a biological specimen. The computer processor manipulates the digital images, and the images are processed to provide a representation of one or more entities that are included within the sample (10843190, 10663712) [75,76].

**Figure 8:**
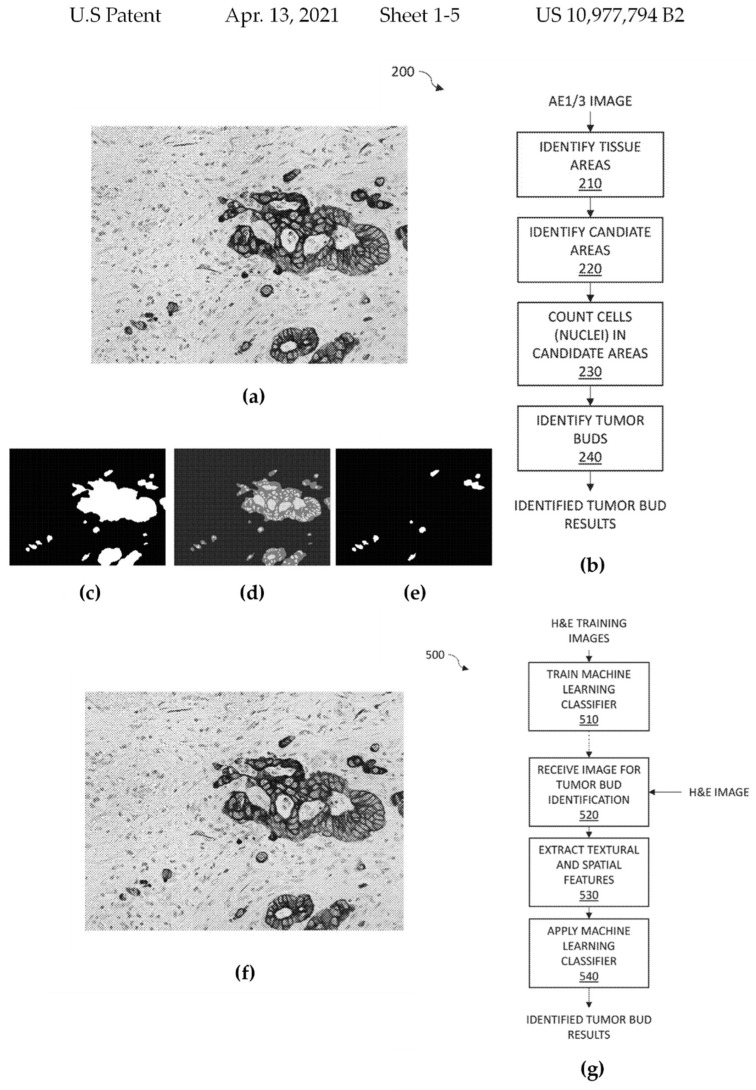
Selected drawings from the patent “Automated identification of tumor buds” (patent 10977794) awarded to the Ohio State Innovation Foundation, Columbus, OH, USA in 2021, (**a**) shows an illustration of a tumor segment stained with pan-cytokeratin AE1/3 under magnification (**b**) flow chart illustrating a method for recognizing tumor buds in an image of a tumor segment stained with pan-cytokeratin AE1/3 (**c**) binary image depicting tissue areas corresponding to the image of figure a (**d**) depicts detected nuclei within the tissue areas (**e**) binary image depicting identified tumor buds (**f**) exemplifies illustrative results of tumor bud identification, wherein the image of figure a is depicted with identifiers of tumor buds (**g**) flow chart illustrating a potential method for recognizing tumor buds in an image of an H&E-stained tumor segment. (reproduced from the public database at www.uspto.gov, accessed on 21 October 2021).

In 2018, the Ohio State Innovation Foundation (Columbus, OH, USA) suggested a disclosure encompassing an image processing and analysis technique to detect tumor buds in an image of a pan-cytokeratin AE1/3-stained segment of a tumor. In this approach, a pixelated image is received. Each pixel is thresholded to determine whether it corresponds to a tissue (e.g., debris such as necrotic tissue). In one implementation of the procedure, the image is quantized by converting it into a grayscale image. An alternative method is to count the pixels inside each detected tissue region to calculate its size. In this manner, tissue regions that are smaller than the lower threshold can be ruled out as potential locations for investigation owing to noise and/or inadequate staining. The nuclei are used as proxies for the cells for cell counting. Each potential region is scanned for nuclei and the discovered nuclei are tallied to determine the cell count. Tumor buds can be detected as clusters with one to five cells. The training method extracts textural/spatial information from each training image and converts these data into vectors for training the ML classifier. Subsequently, tumor sections are graded according to the number of discovered tumor buds and a link between the observed tumor buds and clinical results is determined using regression analysis, as indicated in Figure 8 (10977794) [77].

#### 3.5.8. Annotation

Huron Technologies International, Inc. (St. Jacobs, CA, USA) developed a content-based image retrieval (CBIR) annotation system to identifying an image corresponding to a query image and filed for a patent on 23 September 2016. The system involved a database that has several comparison barcodes that were indexed with their associated comparison images. It was also programmed to compress the plurality of image transform values using an artificial neural network (10628736) [78]. Similarly, in June 2019, Ventana Medical Systems, Inc. (Tucson, AZ, USA) filed a patent based on computer scoring of annotated images based on immunohistochemistry staining. The technique entails the acquisition of first and second images. The first image is an H&E stain, whereas the second image is a biomarker image; by analyzing the first image, attributes are generated from it. Additionally, a computer device comprised of one or more processors and adequate memory processes the first and second image to form a registered image. Later, the biomarkers, such as PD-L1, CD3 or CD8, are analyzed and a probability map is generated based on the first image or the registered image (10977791) [79]. D.R. Systems, Inc. (San Diego, CA, USA) submitted a patent for a system that enables the matching and/or registration of medical imaging examinations to greatly reduce and/or eliminate artifactual differences between the 2D examination images. The newly matched 2D images result in automated 3D registration of the exams and/or multiplanar reformation of 3D volumetric data acquired during one or both tests (e.g., during imaging scans). As a result, the concepts may be used to determine which examinations should be compared and which examinations should be paired. For instance, the system may automatically add indicators to a subsequent acquired image based on an earlier acquired annotated image. Thus, by picking the indications, the physician may be able to easily add and modify equivalent comments on the resulting image. Annotation may include arrows identifying specific places or anatomical structures, circles, polygons, and irregularly shaped sections, among many others. To quantify tumors, vascular stenosis, organs, and other objects, linear dimensions, area, density in Hounsfield units, optical density, volume, and curved lines can be used. (10127662) [80].

## 4. Discussion

In this review study, we obtained information on previous advancements and evaluated the current state of the industry and possible future trends of AI in DP by analyzing patent applications issued by the USPTO, EPO, KIPRIS, CNIPA, and JPO. Patents relating to AI in DP have increased dramatically over the past decade, and the categorization of the inventions revealed that many patents were linked to WSI, segmentation, classification, and detection. Most patents belonged to private enterprises and firms, thereby demonstrating the considerable interest of the private sector in developing AI in DP.

This is the first systematic review offering a patent landscape analysis covering five major patent databases, namely, those of the USPTO, EPO, KIPRIS, JPO, and CNIPA. The findings of this study may be beneficial for countries and organizations that are interested in the development of DP in the area of AI. R&D professionals can use these findings to identify AI applications and approaches that have been studied in R&D efforts worldwide over the past two decades. Such an understanding is critical for governments, academics, and businesses to close the gap in the race to master AI.

Although we covered all major databases, broadening the analysis to include patent data from other patent offices around the world, such as the African Regional Intellectual Property Organization, the Indian Patent Office, the GCC Patent Office, the Federal Service for Intellectual Property, and the World Intellectual Property Organization, could further improve the reliability of the results and should be a consideration for a future study. Moreover, the numerous patent formats, languages, and classification systems that are used by patent offices worldwide make it challenging to locate AI-related patents in DP. For example, in other databases, not all patents are in English, making it difficult to identify AI-related patents using keywords. Furthermore, technological advancements do not always take the form of patents. For example, certain businesses choose not to protect an innovation with a patent because this requires sharing a detailed description of the idea with the public. This characteristic makes it difficult to observe technological trends and advancements by means of patent analysis, which is a limitation of any study based on patent data.

## 5. Conclusions

It can be concluded that the rapid increase in the number of patents pertaining to the use of AI in DP attests to rapid advancement in the field. The main focus areas were WSI, segmentation, classification, and detection. As a result, we foresee a continuous growth in AI in DP and an increase in the number of patent applications internationally. Several applications, such as mutation detection, therapeutic response prediction, and prognosis prediction, are expected to be developed in the future.

## Figures and Tables

**Table 1:** Top 10 inventors and their affiliations.

Inventors	Affiliations	Number of Patents
Fuchs Thomas	PAIGE.AI	25
El-Zehiry Noha	Siemens	25
Arar Nuri Murat	IBM	13
Barnes Michael	Ventana	11
Rusko Laszlo	GE Company	10
Madabhushi Anant	Case Western Reserve Univ.	10
Jianhua Yao	TENCENT	8
Stephen Reserve	TEMPUS LABS	8
Van Driel Marc	Philips	8
Timothy Burton	Analytics For Life	6

## Data Availability

The data presented in this study are available on request from the corresponding author (https://www.researchgate.net/profile/Yosep-Chong, accessed on 3 April 2022) The data are not publicly available due to institutional policies.

## Supplementary Materials

The following supporting information can be downloaded at: https://www.mdpi.com/article/10.3390/cancers14102400/s1, raw data.xls.
